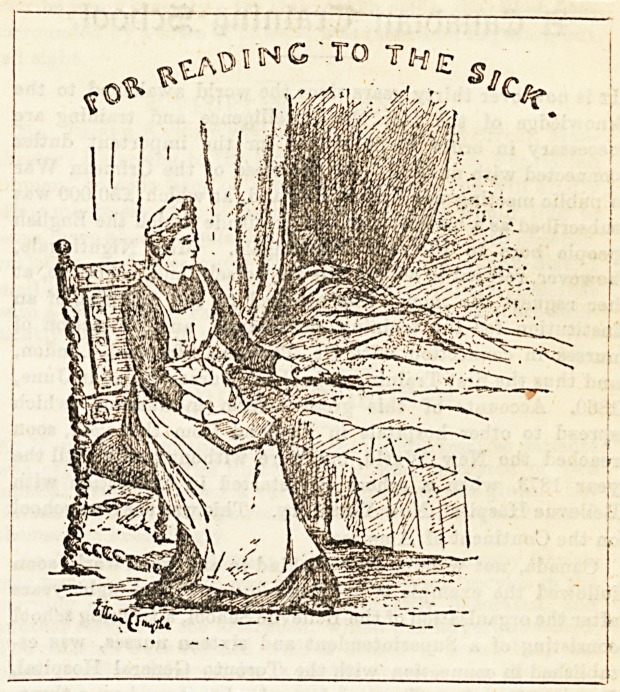# The Hospital Nursing Supplement

**Published:** 1892-01-30

**Authors:** 


"The Hospital" Nursing Mirror.

Being the Extra Nursing Supplement of "The Hospital" Newspaper.

Contributions for this Supplement should be addressed to the Editor, The Hospital, 140, Strand, London, W.C., and should have the word "Nursing" plainly written in left-hand top corner of the envelope.

### En Passant.

FOR THE LEPERS.—We hear that the big box of treasures sent for the children at the Leper Hospital at Colombo arrived safely, and the contents were to be distributed on December 28th, the day on which the Christmas treat was arranged for these poor little folks. The chaplain and his wife were very much pleased by such an acceptable and seasonable Christmas box. We are sorry to find that influenza has made its appearance in Ceylon, and that there are many people in Colombo suffering from this universal epidemic.

"WE NEVER PRESCRIBE."—Again and again we have to give this answer to non-nurse readers who write us long dissertations on their various diseases. This week we have a comical yet typical request for advice: "Leap Year" writes to ask if it is injurious to eat (occasionally) small pieces of coal, and goes on to say that a person who has done so has lately looked rather pale. That "person" is undoubtedly lacking in common sense, and undoubtedly suffering from dyspepsia, and had better consult a medical man at once. But it is extraordinary that there should be so many people in the world who have not the wit to know that medical treatment cannot be given by correspondence, and also pathetic to think that there are so many sufferers who are foolish enough to allow their ailments to become chronic because practically they lack enough ordinary strength of mind to call in a doctor.

THE NURSES' UNION.—The annual social gathering of the Nurses' Union was held at Morley Halls, 316, Regent Street, on January 14th, three to nine p.m. Soon after the hour of invitation a few nurses began to assemble, the numbers increased as the afternoon wore on, until the reading-room looked quite full. The musical part of the programme would be too long to give in detail; it included solos, duets, and trios on the violin, mandolin, zither, and piano, besides several songs and recitations. The nurses were asked to come in uniform, which added to the interest and brightness of the scene; the different costumes looked very picturesque grouped among the green foliage and bright draperies with which the room was decorated. Tea was served in an adjoining room, where the company went in relays. Amongst the guests was an Indian lady who is studying medicine in England. The first speaker was Miss Palethorp, for many years a doctor in India; she gave a very interesting and amusing account of the difficulties of nursing in India. Mrs. Maurice Gregory thrilled all listeners with her graphic words on the opium dens of India, several of which she has herself visited; her description was later amplified by Mr. Puri, a native Bengali gentleman, who reminded those present of the touching message sent by the ladies of India: "If the English ladies will only stop the opium traffic, we will give the skin of our bodies to make their shoes." Mrs. E. W. Moore mentioned her association with the Nurses' Union from its very commencement, and gave some very practical thoughts on the motto for the year: "Watch ye, stand fast in the faith." Miss Orme, the Lady Superintendent of the Temperance Hospital, interested everybody by her bright, edifying speech, which must have stirred many to make their "noble calling" of nursing still more noble and blessed in the practical dealing of daily life. Mr. Albert Carless, Surgeon of King's College Hospital, and the Hon. Louisa Kinnaird also spoke. Mention was made by most of the speakers of the sad death of the Duke of Clarence, which had occurred that morning, and heartfelt sympathy was expressed with the bereaved family.

BEDFORD INSTITUTE.—The report of this Institute which was submitted to a general meeting on the 13th inst., says: "The Council, in presenting their annual report for the year of 1891, have again to draw attention to the large amount of work which has been done by the district nurse. She has during the year had 118 cases and made 2,324 visits. The fact is especially commended to the notice of those who do not as yet subscribe to the funds of the Institute. During the past year the number of private nurses has been raised from nine to twelve. They have attended 131 cases." The accounts are satisfactory, there being a balance of about £900. Further accommodation for the nurses is badly needed, and the town poor would benefit if another district nurse could be provided.

NEW WORK FOR THE BLIND.—Turning in to Mrs. Creighton Hale's the other day we saw a quaint and interesting sight. On the little narrow bed lay a blind girl acting as dummy to a massage class, and one of the members of the class was also blind. Mrs. Hale is teaching these two blind girls free of charge, and hopes to make them skilled masseuses. They both have a wonderfully sensitive touch and a retentive memory. They are chiefly taught by being practised on, an excellent method, for the victim who has felt the difference between the brisk but painless friction given by Mrs. Creighton Hale and the sore and labouring rubbing of a learner of the art knows the first and greatest principle of massage. Of course there are certain nervous cases, where it is necessary to watch the patient's face, that a blind masseuse could never undertake; but in cases of fracture and stiffness, where long and steady manipulation is needed, these two blind girls could do good work. One girl has so far only been able to earn about two shillings a week by knitting, so that it is a grand thing for her to think what an opening is now before her. Anyone willing to employ either of these blind masseuses, and so help forward a good work, should apply to Mrs. Creighton Hale, 151, Oxford Street, W.

SHORT ITEMS.—Subscriptions towards the cross sent for the funeral of the late Duke of Clarence were received from Guy's Hospital, Dublin Jubilee Institute, Nurse Steer, Nurse Frearson, Nurse Herley, and Nurse Geard, in addition to those acknowledged last week. The surplus will be given to the Benevolent Branch of the Pension Fund.—The nurses of the Sheffield Fever Hospital have been in the habit of sending out their uniforms to be made after the material has lain some time in the hospital.—Miss Goldie, for twenty years Matron of the Chalmers Hospital, Edinburgh, has, on her resignation, been granted an annuity.—Last week's *Queen* contains portraits of Dr. Broadbent and Sister Victoria, physician and nurse to the late Duke of Clarence.—The Montrose Queen's nurse has attended 105 cases within the last half-year.—In Edinburgh the applications for the services of the Queen's nurses have lately been far beyond what could be undertaken.—Miss Catton, district nurse for Buxton, received much praise at the late annual meeting of the association.—Sister Rose MacBride sends us a new "Private Nurse's Chart" which is very neat and complete, but she does not mention the price.—A lively correspondence on the religion of the Lady Superintendent of the Norwich Hospital is still going on in the local press.

### Lectures on Surgical Ward Work and Nursing.

By Alexander Miles, M.D. (Edin.), F.R.C.S.E.

### Lecture XLIII.—INSTRUMENTS USED IN LITHOTRITY.

The operation of lithotrity consists in the crushing of a stone inside the bladder, and then removing the fragments by means of a large catheter. It is not applicable to very large or very hard stones, nor is it often performed in very young or very old patients, but when appropriate it is a less serious and simpler operation than that of lithotomy. The presence of a stone in the bladder having been determined by means of one or other of the sounds described under lithotomy, a quantity of tepid boracic lotion is injected into the bladder to ensure that that viscus be full. The instrument used for

Fig. 1a.

Fig. 3a.

Fig. 2a.

crushing the stone is called a lithotrite. It must be of the very best steel, and as large as the urethra will admit of. It consists of two blades, the one sliding inside the other. The outer, or female, blade is fenestrated at the beak, so that the fragments of stone may escape from between the blades, and so prevent clogging of the instrument. The male or inner blade is roughened, or serrated at the beak to catch and then pulverize the stone. The handle consists of a double mechanism, which, on the one hand by means of a sliding spring, catches the stone and holds it firmly between the blades, and on the other, by means of a screw approximates the blades, and so crushes the calculus which is between them. Various patterns of lithotrite are in use, the most popular being that of Thompson and Weiss (Fig. 1a). Bigelow's instrument is heavier, but works on the same principle.

After being crushed to fine fragments the stone has to be removed from the bladder, and this is done by the aid of one or other of the forms of evacuating catheter. The catheters are of large bore, and have a wide eye near the point to admit of fairly large fragments. Some are straight (Fig. 2a),

Fig. 5.

Fig. 6.

and these drain best, while the curved ones are more easily introduced. Fitting these catheters are aspirators, by means of which the fluid is withdrawn from the bladder,

Fig. 4.

Fig. 7.

Fig. 8.

carrying with it the pulverised stone. They are so arranged that the same fluid may be used again and again, each time re-bringing out a quantity of débris, which is prevented entering by certain arrangements of valves and glass receptacles. Examples of these aspirators are Clover's (Fig. 3A), Bigelow's, and Sir Henry Thomson's.

OTHER BLADDER INSTRUMENTS.—*For Tumours of Bladder:* In the diagnosis of vesical tumours, the sounds used in connection with lithotomy may be employed, but now-a-days much more accurate information is obtained by the cystoscope, a hollow instrument, shaped like a sound, carrying in the cavity of its beak a small electric lamp, which is lighted up when in the bladder, and so illuminates the interior of that organ. By the aid of such an instrument, not only can the presence of the tumour be determined, but its shape, size, exact position, and even its nature may be learned. For the removal of such growths the bladder must of course be opened, and then with different instruments the tumours are separated and removed. Fig. 4 shows a scraping instrument used by Sir H. Thompson, and Figs. 5, 6, and 7, three pairs of forceps of different shapes, and with biting edges for the same purpose; Fig. 8 represents a sharp steel loop for scraping away vesical tumours.

The illustrations are used by kind permission of Messrs. Arnold and Sons.

### Lincoln Hospital.

DURING a recent visit to Lincoln one of our staff had an opportunity of seeing over the hospital, which appears to be among the best of those institutions for convenience and salubrity. Built in blocks, after the model of St. Thomas's, it stands on a high table land, but sheltered in some measure by a belt of trees from the too keen blasts which are prevalent there at the equinoxes.

The long colonnades, with their lofty pitch and doors at each end for proper ventilation, render the atmosphere sweet and wholesome, while the broad staircases and shallow steps are equally good for carrying the sad burdens which must frequently go up them, and giving ease to the weak and crippled.

Although not a regular visiting day, we were received with much courtesy by the Matron, who gave us every facility for inspecting the various wards, all of which were eminently clean and comfortable. The cots were snowy white, the long tables had just enough flowers and plants to ornament without encumbering them, and a cheerful fire in the centre of the apartment lit it up most agreeably on this chill October afternoon.

The patients were as contented as could be expected of them, considering their pains and trials, and seemed to fully appreciate the care and attention they received. The children's ward was rather small and dull, but a new one is in course of construction, which will give plenty of light and air to the poor little dots.

The souls of the patients are equally well cared for. The Chancellor of the Cathedral is the responsible Chaplain, but he passes on the salary to another clergyman, who is his zealous coadjutor in the services of the comfortable and reverently-appointed chapel, as well as the personal ministrations to the suffering. The wards also are frequently visited by the wives and daughters of the Cathedral Canons, and are abundantly supplied with books, writing materials, and games for the use and amusement of the sick folk.

We may mention *en passant* that the uniform of the attendants leaves nothing to be desired by either wearer or observer, and the jaunty air with which it and the smart little cap is worn, betokens the nurses' appreciation of it, and also the pride and pleasure she takes in her useful, if at times overburdened, life.

### Presentation.

MRS. FORREST, Matron of the Guernsey Convalescent Home, has been presented by the members of the Mutual Improvement Class she holds with an illustrated Bible.

BROTHERS OF PITY.

"PITY is near akin to love," says the poet, and we know from experience that like love it softens the heart and makes it overflow with sympathy for all who are suffering in either mind or body; yes, pity is a very lovely and tender thing which we may cultivate even when we ourselves are worthy objects of pity to other people.

It is very easy to feel self pity, to think our misfortunes worse than those of others, and to imagine no one has compassion for our feelings or our sufferings. We get into a habit of looking at our own complaints through a magnifying glass and of turning the instrument the reverse way when we inspect our neighbours, in fact making mountains of molehills, and molehills of mountains where it suits our purpose.

Several hundred years ago a number of good men joined together that they might do all kinds of works of mercy for the bodies of their fellow creatures, whether living or dead, and they called themselves "The Brothers of Pity." Like the Good Samaritan they took care of the friendless sick and dying, and buried the bodies of those who had been killed in war or abandoned by their relations. Happily our laws and customs are now so much improved that these necessary duties are undertaken by the State, yet there is plenty of work left us in the world for us, and true Christian pity will point out where it is to be found. Our Divine Master had a heart full of pity while on earth, and has carried that same human heart to Heaven, from whence He looks with pleasure and approval on all who try to follow in His footsteps.

And we may find plenty of opportunities of showing our love and sympathy without moving from our sick couch. We can think of all our relations who are grieving for us and our woes, we can mourn for those who mourn and weep for those who weep. Especially can we pray for them. Just at this time our best feelings should be awakened for the trials of one family, the highest in the land, which has been plunged from the greatest happiness into the lowest depths of woe. Death is no respecter of persons; he has touched with his cold hand the princely boy, who in a few weeks would have a happy bridegroom, but who is now lying with his ancestors. It is a tale full of pathos and regret, the burden of which can only be lightened by prayer. Like true brothers of pity then we will try and forget our own private sorrows and beseech our sympathising Saviour to draw near to His suffering servants in their trouble of mind, to hallow all their crosses in this life, and to crown them hereafter where all tears are wiped away. So shall we be knit in one brotherhood, high and low, rich and poor, under the great pattern for example, our pitying Saviour.

### A Canadian Training School.

It is now over thirty years since the world awakened to the knowledge of the fact that intelligence and training are necessary in order to fit women for the important duties connected with nursing. At the close of the Crimean War a public meeting was held in England, at which £50,000 was subscribed as a memorial of the gratitude which the English people bore to Florence Nightingale. Miss Nightingale, however, refused to accept this for herself, and, therefore, at her request the money was devoted to the founding of an institution for the training, sustenance, and protection of nurses in connection with St. Thomas's Hospital, London, and thus the first Training School for Nurses began in June, 1860. Accounts of this great reform in nursing, which spread to other hospitals in England from that year, soon reached the New World, but were without result until the year 1873, when a school was started in connection with Bellevue Hospital, New York City. This was the first school on the Continent of America.

Canada, not wishing to be behind in any good work, soon followed the example of her sister country, and eight years after the organisation of the Bellevue School, a training school consisting of a Superintendent and sixteen nurses, was established in connection with the Toronto General Hospital, Toronto, Canada. The training school system has a threefold object in view. Its primary and greatest aim, the improvement of the nursing service in the hospital, so that the poor of our community, who would otherwise find it beyond their means, may have every advantage which skilled nursing can provide. Secondly, it aims to be a school of instruction where women who are fitted by nature and education, can obtain a thorough theoretical and practical knowledge of the art of nursing, with a view to making this their calling or profession; and thirdly, it seeks to give the medical profession intelligent and skilful co-operation, in the noble work of alleviating human suffering.

As far as can be ascertained there are at present ten training schools in Canada, but the Toronto School is by far the largest school in the Dominion. It was organised in April, 1881, and has therefore been in operation for ten years. For the last seven years the school has been superintended by Miss Mary A. Snively, a Canadian, who was trained in Bellevue Hospital, New York City. Beginning with sixteen nurses in 1881, in 1891 the school numbered sixty pupil nurses in training, and two permanent nurses, sixty-two in all. The officers of the school consist of a Superintendent, and Assistant Superintendent, one supervising night nurse, and two permanent house nurses—one in the lying-in pavilion and one in the gynæcological pavilion. The uniform of this school is an open brown check skirting, made princess style; white cotton apron with broad strings, tied in large bows at the back; a muslin cap made high in front, and low behind, trimmed with frilling. The head nurses wear the same, only the caps are encircled with a black velvet band. This is the distinguishing mark of a head nurse, and is eagerly sought after, as it is considered an honour to attain to this position.

Every pupil receives three dollars a month, board, washing, and uniforms during the first year; and the second year six dollars per month, with board, &c. This is not looked upon as payment for their services, but is simply to provide them with the necessary text-books, &c. The training school is controlled by the trustees of the hospital, like all the other departments of hospital work, the Medical Superintendent having the general supervision, and the Superintendent of the training school immediate charge of the nursing, course of work, study, and lectures, as well as the discipline of all the nurses in the hospital. It is not customary to admit a class of probationers Spring and Fall, although the examinations are conducted semi-yearly. A nurse may have passed her final examination, yet she remains on duty in the hospital until she has completed a full course of two years.

The training consists of practical work and bed-side teaching, in the hospital, in medical, surgical, gynæcological, obstetrical, eye, ear, and throat wards, together with semi-weekly lectures delivered by the visiting staff and Miss Snively, Lady Superintendent; also classes by the assistant. The lectures are given gratuitously by the most prominent physicians in the city, and embrace a large number of subjects, viz., anatomy, physiology, diseases of the abdominal and respiratory organs, dermatology, contagious diseases, surgical and medical emergencies, obstetrics, gynæcology, and practical nursing. In one of the buildings on the ground, designated "The Pavilion," not only the nursing, but the cooking, is in charge of the nurses. Each nurse spends at least four weeks in the kitchen of this department, and during this time she is able to learn something of the art of cooking for invalids, obtaining a practical, as well as theoretical, knowledge of dietetics.

*(To be continued.)*

### The Nurses' Bookshelf.

The "Ambulance Handbook"* of the St. Andrew's Ambulance Association contains a large amount of useful knowledge. It is far more complete than most volumes of the kind, and has only one fault—it lacks an index. The illustrations are numerous and excellent, and together with the simple word-descriptions make an elementary knowledge of first aid to the injured very easily acquired. We should think the book would be invaluable to the members of ambulance classes throughout the kingdom.

"Notes on Gynæcological Nursing" † is a slim little volume full of information, given in the most crisp and concise style. It is intended for nurses who, being already acquainted with medical and surgical work, are entering on this special line. The first chapter is on cleanliness, the second on observation, the third on manipulation, and the last on special cases. There is an index and an appendix giving the various strengths of antiseptics generally used. A more convenient and practical little book it would be impossible to desire. It contains many directions with regard to minor details which we have never met with elsewhere, while the admirable order and terseness of its construction is itself a valuable lesson in method.

We have also to acknowledge a third edition of Miss Fullagar's pamphlet on "Roller Bandaging,"‡ which is very cheap and fully illustrated.

* Ambulance Handbook, by George T. Beatson, M.D. Published by St. Andrew's Ambulance Association, Glasgow.

† Notes on Gynæcological Nursing, by Dr. Hellier. Churchill. Price 1s. 6d.

‡ A Handbook of Roller Bandaging, by Miss Fullager. Griffith, Farran. Price 6d.

### Everybody's Opinion.

*[Correspondence on all subjects is invited, but we cannot in any way be responsible for the opinions expressed by our correspondents. No communications can be entertained if the name and address of the correspondent is not given, or unless one side of the paper only be written on.]*

### HOSPITAL SCANDALS.

Miss D. Halkin writes: I have read with great interest the wordy warfare that has taken place between you and the editor of the *Pall Mall Gazette*, and cannot in common fairness let pass the opportunity of thanking you for the great help your paper has been in bringing about the late "Eastern Fever Hospital Enquiry," and also in many other respects connected with the same Institution, viz., the improvement in the nurses' food in 1889, brought about through your publishing letters relating to the same. It is true the *Hackney Mercury*, the *Evening News and Post*, and the *Star*, had drawn public attention to the complaints in connexion with the former, while their penny and threepenny contemporaries still held their peace or thought it a subject far beneath their notice, but still there was hesitation to be met with on every side, and it was not until the reading of an extract of your paper at a public meeting of ratepayers that the first resolution was passed which led the Local Government Board to decide upon an enquiry. Your contemporary no doubt means well, but has unfortunately fallen into the same mistake as some of his colleagues; had he read and inwardly digested the leading article in your issue of August 29th last, he might have avoided making such a "pot-pourri" of rate-supported or Poor-law institutions and the great Voluntary Hospitals; as it is one can not help thinking that it is greatly to be regretted that having thus failed to draw a distinction between the two, he has unconsciously (let us hope) prejudiced the public mind against hospitals in general. That he should, after his attention had been called to his mistake, turn round and bestow far from flattering epithets on one who has done so much for the nursing world in general is, most nurses will agree with me, most unpardonable and uncalled for, and shows a weakness of argument. Had your contemporary confined his remarks to some of our upper-ten dailies, or to the truly specialist press, viz., some of the medical papers (one of which, in its issue of December 2nd last, holds me up as a sort of bugbear to the medical profession) which are always ready to side with *La raison du plus fort*, and trying to whitewash authorities, he might have been nearer the mark.

### ON PRESENTATIONS.

"AN OLD MATRON" writes: May I say a few words on the subject of "Presentations." I have for many years held the post of "Matron," and never but once has a presentation been made me, and then the gift (an inkstand) was bought and placed on my table without any knowledge on my part. I was very much averse to accept it, but under the circumstances I did so, at the same time making it perfectly understood, that I would never again receive any gift from my nurses. I have always had a strong feeling on the subject; it is very hard on nurses, who are still sadly underpaid; many, I am sure, would not wish to give, and would not like to be left out; and all have their own relations to whom a little gift at Christmas is a pleasure and a boon. When I have read week by week of "Presentations' I have wondered how Matrons could like to receive the gifts, and it is entirely in their hands to put a stop to them.

### THE NURSES' CROSS.

### A VEGETARIAN DINNER.

MR. JOSEPH KNIGHT writes from 75, Princess Street, Manchester: Referring to the interesting article, "A Vegetarian Dinner," in your issue of 16th inst., perhaps you will kindly allow me to say that if any of your readers would like to learn more about vegetarian food, which is at once cheaper, healthier, and pleasanter than the ordinary mixed diet, I shall be pleased to send a few explanatory papers, together with a few simple vegetarian recipes, to any who are sufficiently interested to write for them.

### Keeping Christmas.

AT NORWOOD COTTAGE HOSPITAL the annual treat to patients was organised by Miss Blanche Grey, and consisted of two musical evenings when a varied programme was given. Dr. Galton and Miss Phillips received the guests, and all enjoyed themselves exceedingly.

At NOTTINGHAM GENERAL HOSPITAL a very happy Christmas was spent. There was early Communion service in the morning at the chapel, which was prettily decorated, and another service in the afternoon, which was largely attended. The patients were regaled with a dinner of roast beef and plum pudding, followed by a choice dessert. In the afternoon and evening those who felt inclined were allowed to smoke in the wards. The dinner was provided by the hospital, and the cake for the tea from the Christmas Tree Fund and by the sisters of the wards. In the evening songs and recitations were given in the wards by the patients and also by members of the medical and nursing staff. In No. 6 Ward a magic lantern entertainment was given by Mr. Blandy. The success of the arrangements for the Christmas festivities were greatly due to the efforts of the Matron, Miss Rimington, who was ably seconded by the members of the medical staff.

Father Christmas visited the CHILDREN'S HOSPITAL, NOTTINGHAM, and gave the small patients such pretty presents as charmed away all pain for the time being. The nurses had decorated the wards, and in the evening they sang hymns to the children and those parents who cared to be present.

At CHALMERS HOSPITAL, Banff, for the first time, an entertainment and dance has been given. The first part of the evening was devoted to a concert, in which Mrs. Gray, the Matron, took part. A pleasing variation in the programme was afforded by a humorous Scotch reading by Miss Watson, a member of the nursing staff. The efforts of the various performers seemed to give much pleasure to the patients, who testified their appreciation by hearty applause. The concert was brought to a close by the company singing the National Anthem. On the conclusion of the first part of the entertainment, the patients received gifts of fruit, and other presents, which had been provided by Mrs. Garden Campbell of Troup; Mr. and Mrs. Ramsay, Earlhill; Mrs. Colvill, The Castle; and Mrs. G. M. Hossack, St. Catherine's. After the patients had retired, the room was cleared for dancing, which was engaged in by the members of the hospital staff and most of the general company. During the evening supper was served in the board-room, and the company separated soon after eleven o'clock, after having spent a very pleasant time.

### Appointments.

HOPE INFIRMARY.—The Guardians of the Salford Union Infirmary, Hope, near Eccles, having decided to appoint a Night Superintendent, Nurse Copland, from the Royal Infirmary, Edinburgh, has been selected for the post, the duties of which she took up on Monday last.

HER MAJESTY has now graciously approved the following Edinburgh nurses being placed on the roll of "Queen's Nurses" for nursing the sick poor in their own homes:—Emma Catherine Nicholson, Ada Annie Donaldson, Adelaide Louisa Eyre, Sarah Keay, Annie Stewart Cameron, Mina Thompson, and Florence May Smith.

### The Little Boy who Did Not Die in a Hospital.

He was not a weakly, long-suffering little child; he did not lie on his bed with a patient angelic expression on his tiny white face. His great brown eyes did not see into the dim future with unchild-like gaze, and his hands did not fold themselves like angels' pinions on his little breast. He did not sing hymns as he should have done, and he never cried for his favourite nurse. He always gave dismal howls when the doctor came round the ward, and instead of saying "99" when they wanted to examine his little chest, he said "No, I won't 'less you give me ha'penny."

He was fond of "ha'pennies" was that little boy, and he seldom came near anyone without asking in a wicked little way for "a ha'penny to buy a stamp to write to his mother who was sick at home." He got heaps of pence, but was never known to write a letter, with one exception, and that time it was sent without a stamp. His little bed was only occupied at night, or when he was sent there for a punishment by a nurse who did not love and cherish him. The said nurse spent much of her valuable time in keeping his fat little hands from doing the small evil deeds that were natural to him.

The neat pad and bandage that should have been on his eye, was occasionally found adorning the cat in a tantalising and quite unsuitable position. While the nurses' scissors which were lost together with a golden curl from his naughty little pate, were discovered, the curl on No. 8's bed, "'cause he was going home and wanted a keepsake," the scissors, in the bathroom, where they had been used to rip open the end of a large bag hung almost out of his reach, to see "what he'd got inside 'im."

His principles were neither good nor high. I should be sorry to say he told stories, because no little boy in a hospital would do that; but he approached as near to them as he possibly could with safety—or perhaps it would be better to say that his chief forte did not lie in the exact understanding or deliverance of a message.

He was sent to the dispensary with a prescription board, and told to carefully carry up the bottle which the dispenser would give him, but he misunderstood, and "carefully" dropped it on the pavement while crossing the square; being sent back with a message to the effect that he was to say he was very sorry he had broken the bottle, he appeared at the dispensary with a serious face, and gravely explained that "Nurse broke the bottle when she was not looking at the steps by the ward door, and fell down, and she wanted another quickly 'cause she was afraid the doctor would scold her, and if the dispenser would give it she would never, never, long as she lived, break medicine bottles again."

His search for knowledge was great; one day a porter discovered him lying flat on the ground trying to peer beneath the grating of the mortuary door, and another time the housekeeper suffered at his hands. He wandered into her room, during her absence, turned her inkstand upside down on the table, and completely obliterated many hours' work in an account book with sprawling representations of inky fingers. He strolled forth at last with his hands behind his back, and a long cherished and only blossom from the window-plant, gaily reposing in the sailor collar of his dirty little jacket.

His favourite song, "Here they come, Fife and Drum, see the Lads in Red," could be heard at all times, especially when silence was required. As a drum accompaniment to his singing he would bang his small fists on anything conveniently near in a way that provoked instant dismissal to a more secluded spot. He was discharged at last to everybody's intense relief, but alas! two days later "the lads in red" sounded as gaily as ever as he marched into the ward. He broke off in the middle of his refrain to make a frantic dash at the nurse to ask if she was not glad to have him back. And it cost that nurse a shilling to pay his tram fare and send him home in charge of the conductor. His anxious friends meanwhile had been searching high and low for him, as he had given them no hint of his intended visit to the hospital, knowing too well they would not let him come such a long way alone.

Even now he is not entirely got rid of, for he turns up occasionally with the remark that he is come to spend the day, and his big brother will fetch him home in the evening.

I wish some of those good little boys who don't give any trouble would come to our hospital.

### Where to Go.

On February 2nd, 3rd, 4th, and 5th, Dr. Symes Thompson will deliver the Gresham Lectures on "Physic" at 6 p.m. at Gresham College, Basinghall Street, admission free.

At South Kensington Museum Saturday lectures are delivered at three o'clock, admission 1s. February 13th, 20th, and 27th., the Rev. George Forrest Browne, B.D., F.S.A., Canon of St. Paul's, Disney, Professor of Archæology at Cambridge, "Early Christian Art: (1) Ireland, (2) Scotland and Man, (3) England."

Miss Jane Harrison commences a course of three lectures on Greek Art at South Kensington Museum on February 3rd. Ticket for the course, 10s. 6d.

On February 10th, Mr. Maurice Hewlett commences at South Kensington a course of six lectures on "The Revival of Platonism." Tickets for the course, one guinea.

At Essex Hall Strand, on February 5th, at eight o'clock, Mr. Massingham will lecture on "The Method of Fabianism." On February 19th, Mrs. Grenfell will lecture on "Socialism and Women," admission free.

Sunday Lecture Society.—The third series of lectures given by this society begins on Sunday afternoon, the 31st of January, in St. George's Hall, Langham Place, at four o'clock, when Mr. Sergius Stepniak will lecture on "Count Tolstoi, the Russian Novelist and Social Reformer, the Prophet of a New Religion." Lectures will subsequently be given by Dr. Andrew Wilson, F.R.S.E.; Mr. George Wotherspoon; Mrs. Proctor (widow of the late Richard A. Proctor, F.R.A.S.); Mr. Frank Kerslake, Miss Amelia B. Edwards, and Dr. E. E. Klein, F.R.S.

### Notes and Queries.

### Answers.

*Bamboo Screens.*—Can be had from Jenks and Wood.

*Nurse Alice.*—Ask your medical man; electricity should never be used save under medical advice.

*A Pro.*—Wear woollen stockings, and rub your feet with methylated spirit, or Balm of Bethesda. To pour methylated spirit into your shoes when your feet are very bad gives immediate relief. But time alone will enure you to all the hardships of ward work.

*A Nurse.*—The book you mention is too technical and medical; you would probably find "The Management of Children" (published by Churchill) or "Our Baby," by Mrs. Hewer, more suitable. Both books have somewhat to say on the wasting diseases of childhood.

*M. S. R.*—We know of no book specially devoted to fever nursing, but there are good chapters on that subject in Lewis's "Practice and Theory of Nursing," price 3s., from this office, or in Norris's "Nursing Notes."

*Sister Edith.*—If you have served three years in a good general hospital, apply to the Director-General, Army Medical Department, Whitehall, S.W., and say you wish to become one of Her Majesty's Nursing Sisters, and would prefer foreign service.

*Influenza Case.*—Mrs. Power, St. Luke's Home, 29, Upper Montague Street, Montague Square, W., will take in and nurse for a moderate charge cases of influenza such as that described by *J. Knight.*

*Lady Superintendent.*—There was not time to write to all the Persion Fund nurses, and the first few letters brought back a more than sufficient response. Thanks for your letter.

*The Nurses' Bed.*—Two shillings received from Nurse A. B.

*Stella.*—Take two yards of flannel, fold it longways; six inches from the fold at the top sew the two sides together (this makes a sort of hood at the back); twelve inches from the hood sew to each side a ribbon for tying in front, two other strings rather lower. Turn back slightly the two points of the lower side, and stitch each together where turned back (this forms the sleeves).

## Figures and Tables

**Fig. 1a. f1:**
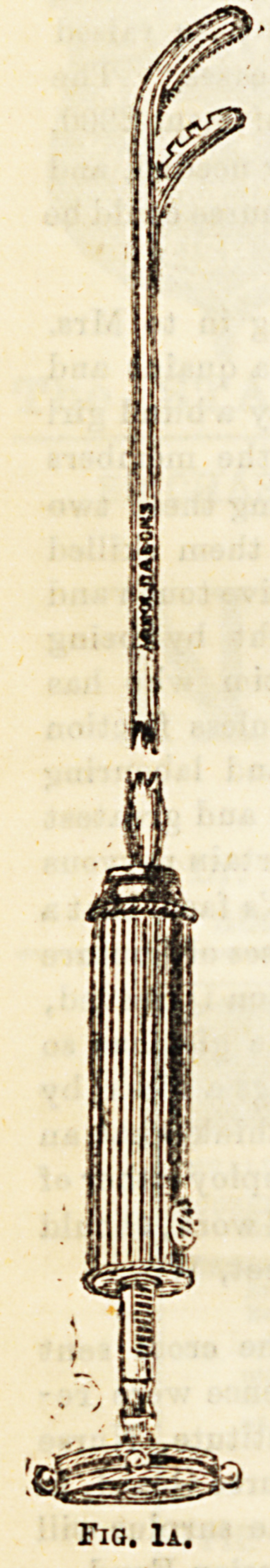


**Fig. 3a. f2:**
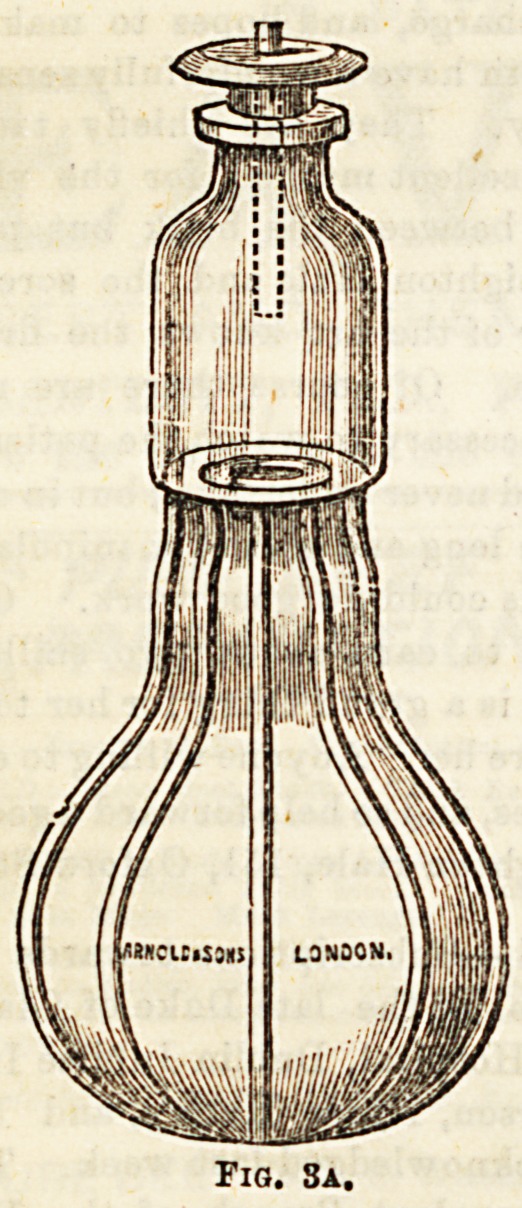


**Fig. 2a. f3:**
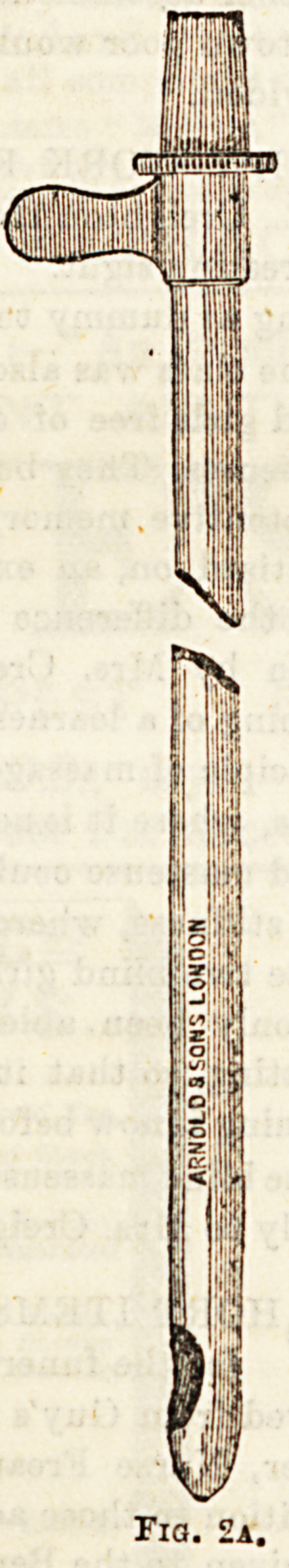


**Fig. 5. f4:**
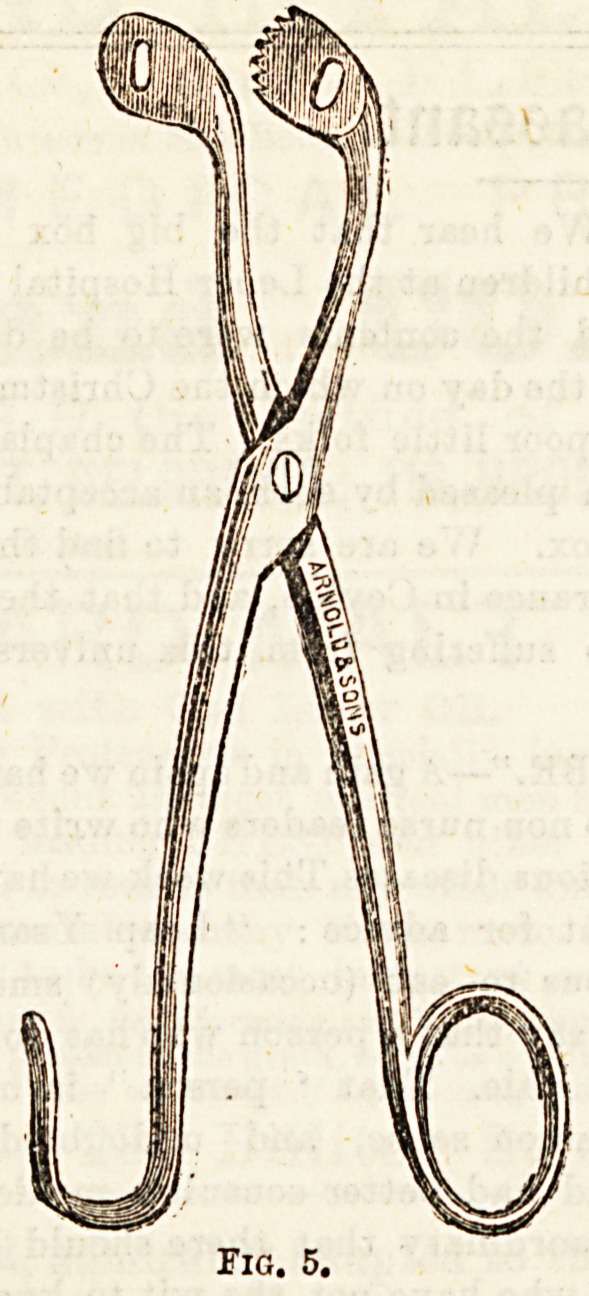


**Fig. 6. f5:**
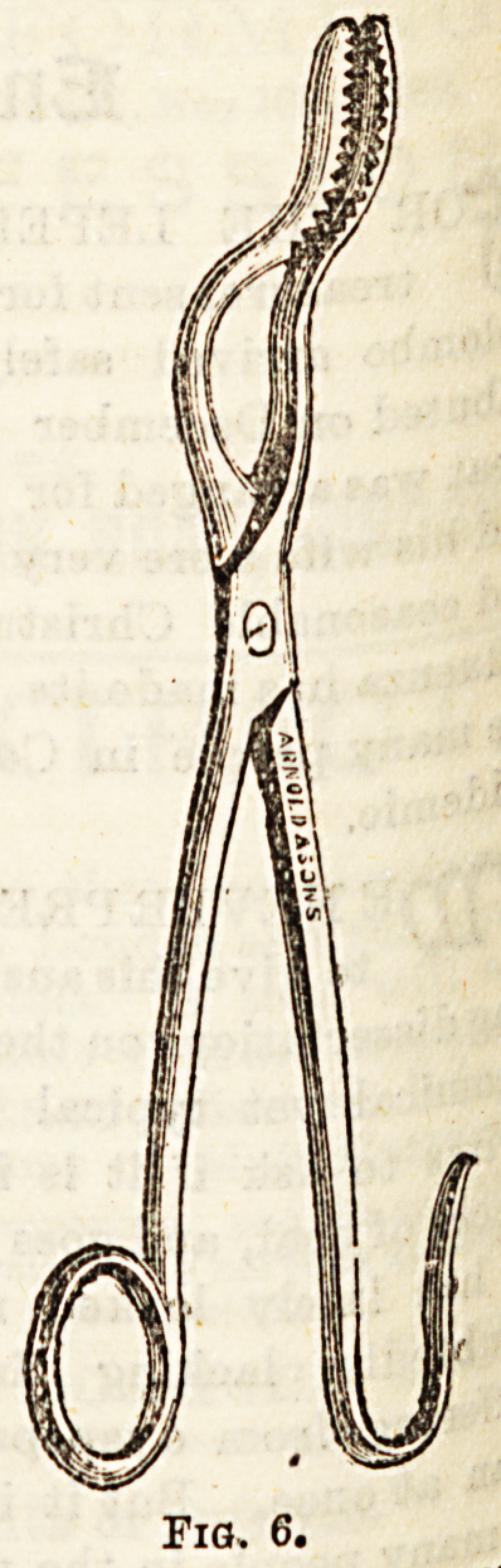


**Fig. 4. f6:**
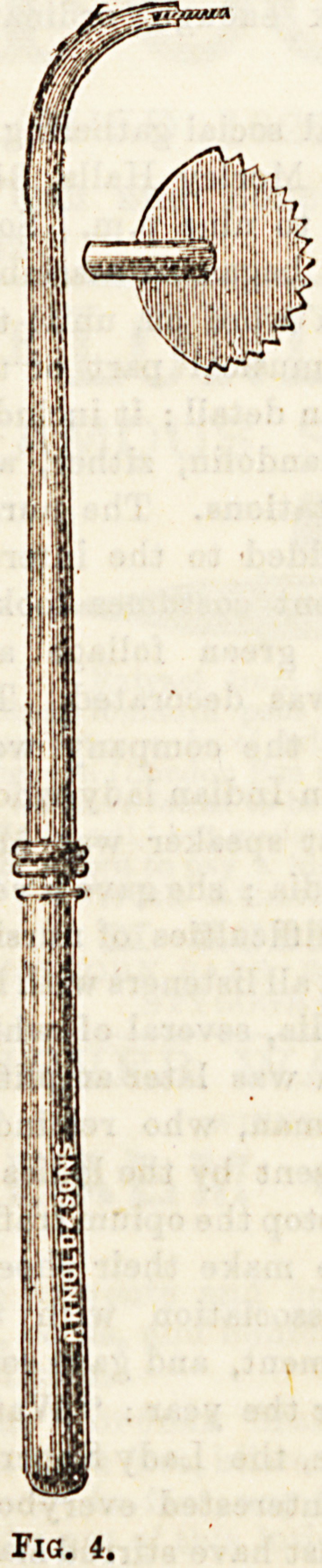


**Fig. 7. f7:**
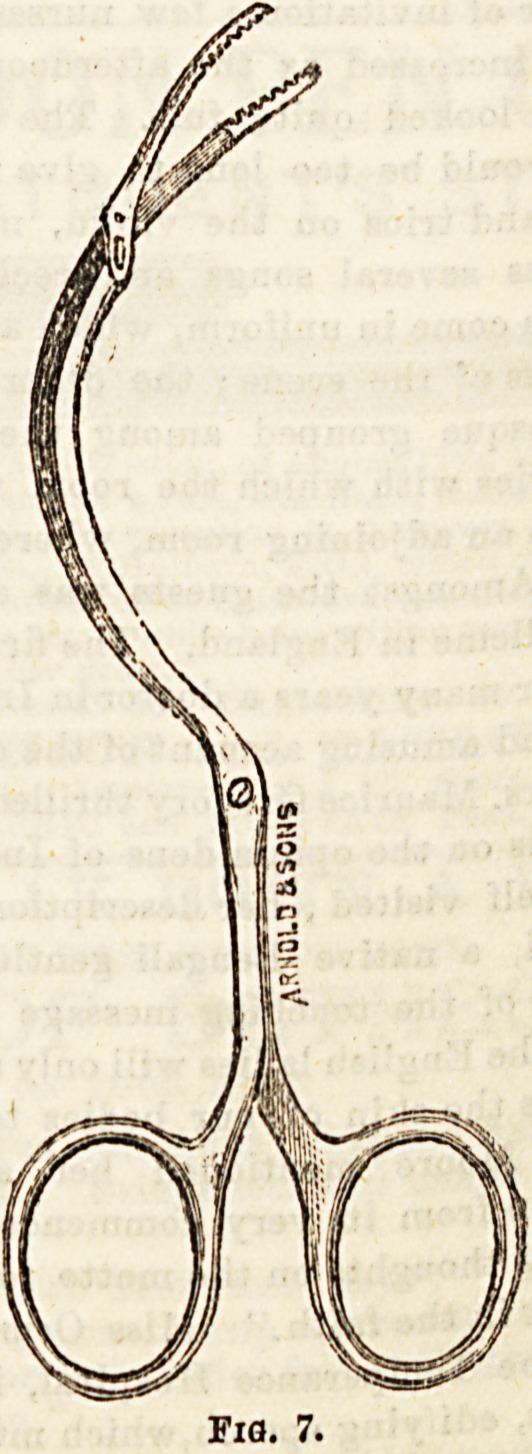


**Fig. 8. f8:**
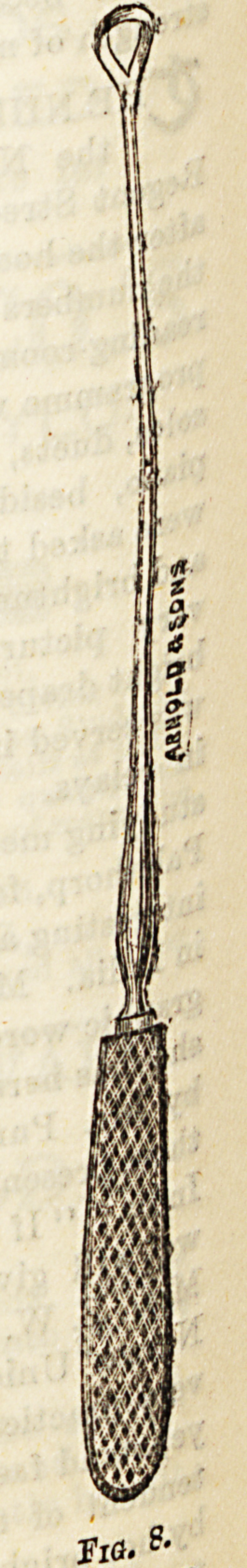


**Figure f9:**